# Relationship between oxidative stress and endometrial polyps in pre-and postmenopausal women

**DOI:** 10.12669/pjms.41.1.10170

**Published:** 2025-01

**Authors:** Emre Erdem Tas, Eyup Ozgen, Gamze Yilmaz, Almila Senat

**Affiliations:** 1 Emre Erdem Tas, Department of Obstetrics and Gynecology, Republic of Turkey Ministry of Health, Ankara City Hospital, Turkey; 2 Eyup Ozgen, Department of Obstetrics and Gynecology, Guven Hospital, Ankara, Turkey; 3 Gamze Yilmaz, Department of Obstetrics and Gynecology, Republic of Turkey Ministry of Health, Ankara City Hospital, Turkey; 4 Almila Senat, Department of Biochemistry, Republic of Turkey Ministry of Health, Taksim Training and Research Hospital, Istanbul, Turkey

**Keywords:** Endometrium, Polyps, Oxidants, Antioxidants, Thiols

## Abstract

**Objective:**

This study aimed to investigate the relationship between oxidative stress (OS) and endometrial polyps (EP) in pre- versus postmenopausal women with abnormal uterine bleeding.

**Methods:**

This prospective case control study was conducted in the Gynecology Department of Ankara Bilkent City Hospital between January and December 2019. In this study, the EP and control groups included 45 participants each (30 pre- and 15 postmenopausal women). Demographics (age, gravidity, parity, and body mass index), serum complete blood count parameters, serum total antioxidant status, total oxidant status (TOS), oxidative stress index (OSI), and thiol/disulfide balance were compared between groups. Subsequently, all women were stratified based on menopausal status, and the investigated parameters were investigated separately in the pre- and postmenopausal participants between groups.

**Results:**

There were no significant intergroup differences in the investigated parameters among all participants (P>0.05). However, unlike in the control group, the age distribution in the EP group was bimodal, peaking in the early 40s and late 50s. Stratification based on menopausal status revealed no significant intergroup differences in the investigated parameters among the premenopausal participants. However, among the postmenopausal participants, serum TOS and OSI levels were significantly higher in the EP versus control group (9.0 vs. 7.2 μmol H_2_O_2_ Equiv/L and 0.8 vs. 0.6 AU; P=0.01).

**Conclusions:**

Serum OS may play a role in the development of EP, particularly among postmenopausal women. Further investigations are required in this area.

## INTRODUCTION

Endometrial polyps (EP) are focal endometrial outgrowths containing variable amounts of glands, stroma, and blood vessels. The prevalence of EP is reportedly 8–35% and increases with age.^1^ They can be asymptomatic or manifest as symptoms such as abnormal uterine bleeding (AUB) (i.e., heavy menstrual bleeding, intermenstrual bleeding, and irregular menses), infertility, and premalignant and malignant status. Common risk factors include obesity, late menopause, advanced age, hypertension, tamoxifen use, and estrogen use without progesterone.^1^,^2^

Numerous molecular mechanisms have been suggested to play a role in EP development, including endometrial aromatase overexpression, monoclonal endometrial hyperplasia, genetic mutations, and endometritis.^3^,^4^ However, the exact cause of EP remains unknown, and their heterogeneity makes the identification of a single causative factor unlikely. Oxidative stress (OS), caused by external and internal factors, was recently suggested to play a role in the development and maintenance of epithelial polyps, leading to uncontrolled cellular proliferation.^5^,^6^

In normal cellular processes, a certain amount of ROS and/or nitrogen species is produced, which is typically balanced by the body’s antioxidant defense system. However, if excessive amounts of these reactive species are generated, they can overwhelm the antioxidant defenses, resulting in OS. This can create an environment that is harmful to the normal physiological functions of women.^7^ Many oxidants and antioxidants reflect OS within an organism. However, measuring the individual levels is both challenging and expensive. In the 2000s, a novel automated colorimetric method for measuring total antioxidant status (TAS), total oxidant status (TOS), and oxidative stress index (OSI) was developed by Erel that provides an overall evaluation.^8^,^9^

A few years later, Erel and Neselioglu developed a new method of analyzing the dynamic thiol/disulfide balance (TDB) that allows for the measurement of serum OS in a different way.^10^ Thiols, characterized by the presence of a sulfhydryl group, serve as defense mechanisms against the detrimental effects of reactive oxygen species (ROS), for which they are the primary targets, as oxidant molecules oxidize them to form reversible disulfide bridges. TDB is essential for various physiological processes, such as antioxidant defense mechanisms, apoptosis, xenobiotic detoxification, cellular signal transmission, and protein stabilization.^11^ Only a few studies to date have investigated the relationship between OS and EP. However, their results remain controversial.^3^,^6^,^12^,^13^ This study aimed to examine the relationship between EP and OS by measuring TAS, TOS, OSI, and TDB in pre- and postmenopausal women with AUB.

## METHODS

This prospective case-control study was conducted at the Gynecology Department of Ankara Bilkent City Hospital between January and December 2019. The sample size was calculated for a total of 90 cases (45 women per group), providing 96% power when a value of α=0.05 was used. G*Power ver. 3.0 Software® (Germany) was used for calculations.

### Ethical Approval:

This study was conducted in accordance with the Declaration of Helsinki and approved by the Ethics Committee of Ankara Yildirim Beyazit University on December 11, 2018 (approval number: 122). Written informed consent was obtained from all participants prior to their participation.

The study participants were women with AUB admitted to gynecology clinics who underwent hysteroscopic examinations. Endometrial samples were obtained from all patients and the EP removed using hysteroscopy. The EP group comprised patients diagnosed with EP on hysteroscopy and pathological analyses. The control group included patients diagnosed with various benign endometrial conditions, such as proliferative, secretory, irregular, and atrophic endometria but without endometritis or EP.

### Exclusion Criteria:

Patients with endometrial hyperplasia or endometrial carcinoma; smokers; alcohol drinkers; hormone replacement therapy users; and individuals with thyroid disorders, polycystic ovary syndrome, rheumatological or adrenal diseases, hepatic or renal failure, diabetes, hypertension, or acute or chronic inflammation were excluded from the study. Furthermore, we excluded patients with severe anemia (hemoglobin levels < 10 g/dL), leukocytemia (white blood cell [WBC] count > 12 (×10^3^/μL), or obesity (body mass index [BMI] > 30 kg/m^2^) to avoid any adverse effects on OS parameters. Blood samples were collected into ethylenediaminetetraacetic acid tubes for complete blood count (CBC) determination and pure tubes for serum TAS, TOS, and TDB parameters before the hysteroscopy.

The CBC determination was made within one hour using a Symex XE-2100 automatic complete blood count analyzer (Sysmex Europe, Germany). The serum WBC count (×10^3^/μL), hemoglobin level (g/dL), and platelet count (×10^3^/μL) were recorded for all cases. Other blood samples were separated into serum samples by centrifugation at 3200 rpm for 10 minutes. The separated serum samples were stored at -80°C until analyzed at the end of the study for TAS, TOS, and TDB levels. Serum TAS and TOS levels (expressed as mmol/L Trolox Equiv/L and μmol H_2_O_2_ Equiv/L, respectively) were measured using commercially available kits (Rel Assay Diagnostics, Turkey).

The OSI was determined using the TOS/TAS ratio. To calculate this, the TAS value was converted to μmol/L, while the OSI value was determined using the following formula: OSI (arbitrary units) = TOS (μmol H_2_O_2_ Equiv/L)/TAS (μmol Trolox Equiv/L). The levels of plasma thiols, including native thiol (NT), disulfide (DS), and total thiol (TT) (NT + DS), were measured using a fully automated method developed by Erel and Neselioglu, with the results expressed in μmol/L.^10^ The DS/NT, DS/TT, and NT/TT ratios (%) were calculated for each participant.

After all necessary data were gathered, the EP and control groups were compared in terms of demographics (age, parity, gravidity, menopausal status, and BMI [kg/m^2^]), CBC parameters (WBC count, hemoglobin level, and platelet count), and serum TAS, TOS, OSI, and TDB levels. Subsequently, all women were stratified by menopausal status, and the EP and control groups were compared separately in terms of the investigated parameters of the pre- and postmenopausal participants.

### Statistical analyses:

The statistical analyses were performed using SPSS (version 21.0; IBM Corp., Armonk, NY, USA). The Kolmogorov-Smirnov test was used to assess data normality. Normally distributed data are expressed as mean±standard deviation, whereas nonparametric data are presented as median and interquartile range. The independent samples t-test and Mann-Whitney U test were used to compare parametric and nonparametric data, respectively, between the groups. Categorical variables are expressed as number and percentage, and the groups were compared using the chi-squared test. Statistical significance was set at P≤0.05.

## RESULTS

**Fig. 1:**
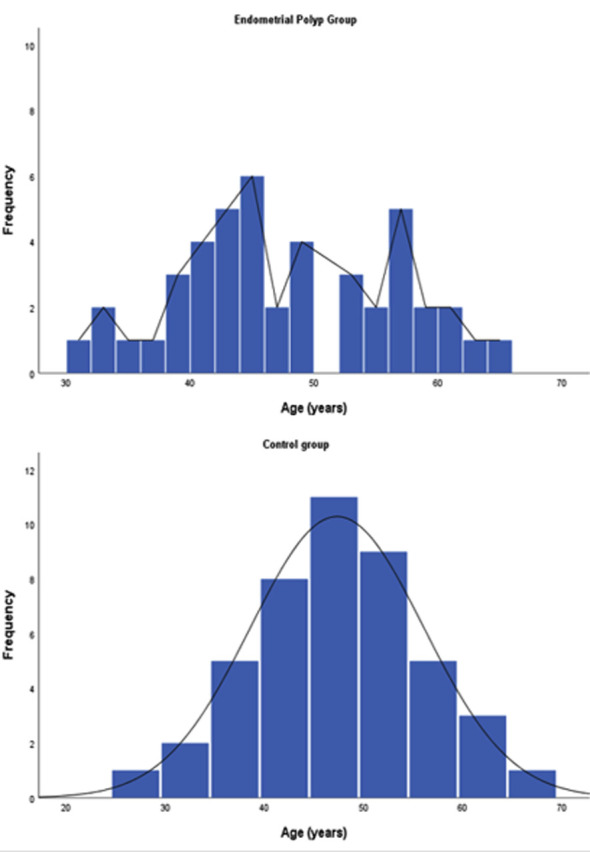
Age distributions of the endometrial polyps and control groups

The EP and control groups of this study included 45 participants each (30 pre- and 15 postmenopausal women). When all women were considered, there were no significant differences in age, gravidity, parity, menopausal status, BMI, CBC parameters, or serum levels of TAS, TOS, OSI, or TDB between the EP and control groups (P>0.05). However, age distribution in the EP group was bimodal, unlike that in the control group (Fig.1). The demographic characteristics, CBC parameters, and serum levels of TAS, TOS, OSI, and TDB in the EP versus control groups are summarized in Table-I.

**Table I:** Demographic characteristics, CBC parameters, and serum levels of TAS, TOS, OSI, and TDB in the endometrial polyps and control groups.

Characteristic	Endometrial polyps group (n = 45)	Control group (n = 45)	P
Age (years)	47.2 ± 8.7	47.3 ± 8.8	0.97
Gravidity	3 (2)	3 (2)	0.16
Parity	2 (2)	2 (1)	0.06
BMI (kg/m^2^)	28.6 ± 4.8	29.1 ± 4.4	0.58
***Menopausal status***			
Post-menopausal	15 (16.7)	15 (16.7)	1.0
Pre-menopausal	30 (33.3)	30 (33.3)	
WBC (×10^3^/μL)	7.1 ± 1.7	6.9 ± 2.3	0.67
Hemoglobin (g/dL)	12.0 ± 1.4	12.3 ± 1.1	0.23
Platelet count (×10^3^/μL)	300.0 ± 71.0	292.5 ± 72.9	0.62
Serum TAS level (mmol/L Trolox Equiv/L)	1.3 ± 0.1	1.1 ± 0.1	0.10
Serum TOS level (μmol H_2_O_2_ Equiv/L)	8.2 (3.1)	9.5 (3.4)	0.56
OSI (AU)	0.8 ± 0.3	0.9 ± 0.4	0.27
Serum NT level (µmol/L	419.8 ± 42.1	414.0 ± 44.3	0.21
Serum DS level (μmol/L)	15.1 ± 5.7	15.5 ± 5.4	0.21
Serum TT level (µmol/L)	449.9 ± 43.2	445.6 ± 44.7	0.70
Serum DS/TT Ratio (%)	3.3 ± 1.2	3.5 ± 1.2	0.56
Serum DS/NT Ratio (%)	3.6 ± 1.3	3.8 ± 1.5	0.43
Serum NT/TT Ratio (%)	94.4 ± 5.2	92.8 ± 2.9	0.08

Data are presented as mean ± standard deviation, median (interquartile range), or n (%). AU, arbitrary unit; BMI, body mass index; CBC, complete blood count; DS, disulfide; IQR, interquartile range; NT, native thiol; OSI, oxidative stress index; SD, standard deviation; TAS, total antioxidant status; TOS, total oxidant status; TT, total thiol; WBC, white blood cell.

The stratification of all participants based on menopausal status revealed no significant intergroup differences in demographics, CBC parameters, or serum levels of TAS, TOS, OSI, and TDB among the premenopausal participants (P>0.05). Similarly, no significant intergroup differences were noted in demographics, CBC parameters, or serum TAS and TDB levels among the postmenopausal participants (P>0.05). However, serum TOS and OSI levels were significantly higher in the EP versus control group among postmenopausal participants (9.0 vs. 7.2 μmol H_2_O_2_ Equiv/L and 0.8 vs. 0.6 AU; P=0.01). Demographics, CBC parameters, and serum levels of TAS, TOS, OSI, and TDB of the EP and control groups after stratification according to menopausal status are shown in Table-II.

**Table II:** Demographic characteristics, CBC parameters, and serum levels of TAS, TOS, OSI, and TDB in the endometrial polyps and control groups after stratification by menopausal status.

Characteristic	Premenopausal Women (n = 60)			Postmenopausal Women (n = 30)		

	Endometrial polyps group (n = 30)	Control group (n = 30)	P	Endometrial polyps group (n = 15)	Control group (n = 15)	P
Age (years)	42.0 ± 5.2	42.8 ± 6.2	0.62	57.6 ± 3.6	56.3 ± 5.4	0.45
Gravidity	2 (2)	2 (1)	0.36	3 (2)	3 (1)	0.23
Parity	2 (2)	3 (2)	0.28	2 (1)	2 (1)	0.98
BMI (kg/m^2^)	28.4 ± 5.0	30.6 ± 4.6	0.68	28.7 ± 4.4	29.3 ± 4.0	0.72
WBC (×10^3^/μL)	7.1 ± 1.9	7.3 ± 2.4	0.84	7.1 ± 0.9	6.3 ± 2.1	0.21
Hemoglobin (g/dL)	12.3 ± 0.9	12.5 ± 0.8	0.31	11.8 ± 1.5	12.2 ± 1.3	0.31
Platelet count (×10^3^/μL)	282.2 ± 65.0	296.1 ± 84.2	0.48	315.8 ± 66.0	286.4 ± 44.0	0.12
Serum TAS level (mmol/L Trolox Equiv/L)	1.1 ± 0.1	1.1 ± 0.1	0.17	1.1 ± 0.1	1.1 ± 0.1	0.36
Serum TOS level (μmol H_2_O_2_ Equiv/L)	9.8 (5.9)	9.7 (5.0)	0.94	9.0 (1.4)	7.2 (1.3)	0.01
OSI (AU)	0.9 ± 0.5	0.8 ± 0.3	0.73	0.8 ± 0.1	0.6 ± 0.1	0.01
Serum NT level (µmol/L)	433.3 ± 30.9	420.7 ± 40.0	0.17	375.3 ± 42.4	365.0 ± 30.6	0.45
Serum DS level (μmol/L)	15.3 ± 5.3	13.2 ± 5.4	0.12	15.8 ± 5.8	15.9 ± 4.3	0.95
Serum TT level (µmol/L)	463.9 ± 34.7	447.1 ± 40.3	0.09	409.0 ± 40.6	386.4 ± 48.5	0.18
Serum DS/TT Ratio (%)	3.3 ± 1.0	2.9 ± 1.2	0.25	3.8 ± 1.5	4.1 ± 0.8	0.63
Serum DS/NT Ratio (%)	3.6 ± 1.2	3.2 ± 1.3	0.12	4.3 ± 1.8	4.4 ± 0.9	0.96
Serum NT/TT Ratio (%)	93.4 ± 2.4	94.0 ± 2.3	0.33	91.7 ± 3.5	95.1 ± 8.5	0.15

Data are presented as the mean ± standard deviation or median (interquartile range). AU, arbitrary unit; BMI, body mass index; CBC, complete blood count; DS, disulfide; IQR, interquartile range; NT, native thiol; OSI, oxidative stress index; SD, standard deviation; TAS, total antioxidant status; TOS, total oxidant status; TT, total thiol; WBC, white blood cell.

## DISCUSSION

EP is a common condition that affects women across age groups, especially those nearing menopause, and constitutes approximately 15% of cases of AUB.^14^-^16^ This condition is considered related to hormonal imbalances during the perimenopausal transition.^2^,^17^ However, previous research indicates the potential for a connection between OS and both benign and malignant gynecological conditions as well as adverse obstetric outcomes.^18^ In recent years, efforts have been made to determine whether a relationship exists between OS and EP.^3^,^6^,^12^,^13^ Our findings suggest that OS may play a role in the development of EP, particularly among postmenopausal women.

In this study, the mean patient age in the EP and control groups was 47 years, which aligns with previous research.^14^,^19^ However, our limited data show that the occurrence of EP may be bimodal, peaking in the early 40s and late 50s, unlike that in the control group. Similarly, in previous retrospective reports, the mean and median ages of pre- and postmenopausal women with EP were approximately 40 and 60 years, respectively.^20^-^22^ This suggests that factors other than hormones may play a role in EP development.^3^,^4^

In the present study, serum levels of TAS, TOS, and OSI did not differ significantly between the EP and control groups among the cohort with AUB. To the best of our knowledge, Pejić et al. were the first to investigate the relationship between OS and EP.^13^ They measured serum antioxidant status using serum superoxide dismutase, glutathione reductase, glutathione peroxidase, and catalase enzymes as well as serum antioxidant status using lipid hydroperoxide levels. While the serum levels of superoxide dismutase and glutathione reductase were significantly lower, those of glutathione peroxidase were significantly higher in patients with EP versus healthy controls. However, no significant differences were observed between the serum levels of catalase and lipid hydroperoxide. In another study, Cinar et al. measured serum antioxidant status using catalase and xanthine oxidase enzymes, as well as serum oxidant status using malondialdehyde levels.^6^ They found that the serum levels of antioxidants and oxidants were significantly higher in patients with EP versus healthy controls. Later, Nayki et al. investigated the serum levels of TAS and TOS using a novel method developed by Erel.^8^,^9^,^12^ Similar to our findings, the authors found that the serum levels of TAS, TOS, and OSI did not differ between patients with EP and healthy controls. We attribute the difference between our results and those of Nayki et al. versus those of Pejić et al. and Cinar et al. to methodological differences in serum OS level assessments. However, we believe that measuring OS markers collectively rather than individually is more practical and inexpensive yet comparable.

Unlike previous studies, the relationship between OS and EP was also examined in this study according to menopausal status. Our findings revealed that serum levels of TOS and OSI were significantly higher in postmenopausal women with EP. Unlike in premenopausal women, the absence of hormonal factors in postmenopausal women suggests that there may be different factors in the etiology of postmenopausal EP. Our findings suggest that OS may play a role in the development and maintenance of EP, particularly among postmenopausal women. To the best of our knowledge, this is the first study to examine the relationship between EP and OS according to menopausal status.

Thiols are essential antioxidants that help eliminate ROS nonenzymatically, and maintaining the TDB is crucial for some detoxification processes. Previous studies highlighted parameters such as NT, DS, and TT levels as well as DS/TT, DS/NT, and NT/TT ratios that are important for TDB.^3^,^10^,^23^ Ozaksit et al. previously investigated the relationship between TDB and EP and found that TDB levels did not differ between patients with EP and healthy controls.^3^ Similar to the study by Ozaksit et al., the present study found no significant differences in TDB between the EP and control groups among the AUB cohort. Furthermore, this finding did not differ after stratification of the participants based on their menopausal status.

### Limitations:

A significant limitation of this study was its limited sample size due to the limited availability of biochemical kits for the TAS, TOS, and TDB measurements. Therefore, we could not investigate the role of OS in different benign, premalignant, and malignant endometrial pathologies or compare their serum levels with those of women with EP. However, we hope that our findings provide a new perspective on the relationship between OS and EP and offer guidance for future research.

## CONCLUSION

This study is the first to investigate the relationship between OS and EP in pre- and postmenopausal women with similar demographic characteristics. Our findings revealed that EP peaked in the early 40s and late 50s, and serum TOS and OSI levels were significantly higher in postmenopausal women with EP versus controls. This finding suggests that OS may play a role in the development of EP, particularly among postmenopausal women. Further investigations are required in this area.

### Author Contributions:

**EET and EO:** Study hypothesis, methods design, data collection and processing, literature search, and article writing and editing.

**GY:** Methods design, study organization and monitoring, data collection, critical eanalysis and preparing the manuscript.

**AS:** Organizing and execution of the project, monitoring its progress.

All authors have read the final versio and are accountable for the integrity of the study.
